# New bakuchiol dimers from *Psoraleae Fructus* and their inhibitory activities on nitric oxide production

**DOI:** 10.1186/s13020-021-00499-y

**Published:** 2021-10-07

**Authors:** Qingxia Xu, Qian lv, Lu Liu, Yingtao Zhang, Xiuwei Yang

**Affiliations:** State Key Laboratory of Natural and Biomimetic Drugs, Department of Natural Medicines, School of Pharmaceutical Sciences, Peking University Health Science Center, Peking University, No. 38, Xueyuan Road, Haidian District, Beijing, 100191 China

**Keywords:** *Psoralea corylifolia*, bakuchiol dimers, Inhibition of nitric oxide

## Abstract

**Background:**

Dried fruits of *Psoralea corylifolia* L. (*Psoraleae Fructus*) is one of the most popular traditional Chinese medicine with treatment for nephritis, spermatorrhea, pollakiuria, asthma, and various inflammatory diseases. Bakuchiol is main meroterpenoid with bioactive diversity from *Psoraleae Fructus*. This study was designed to seek structural diverse bakuchiol derivants with anti-inflammatory activities from this plant.

**Methods:**

Various column chromatography methods were used for isolation experiment. Structures and configurations of these compounds were determined by spectroscopic methods and single-crystal X-ray diffraction. Their inhibition on nitric oxide (NO) production in lipopolysaccharide (LPS)-stimulated RAW264.7 macrophages were evaluated by the Griess reaction.

**Results:**

Twelve unpresented bakuchiol dimmers, bisbakuchiols M–U (**1**–**9**) and bisbakuchiol ethers A–C (**10**–**12**), along with five known compounds (**13**–**17**), were isolated from the fruits of *Psoralea corylifolia* L. Compounds **1**–**3**, **10**–**12**, **16** and **17** exhibited inhibitory activities against LPS-induced NO production in RAW264.7 macrophages, and the inhibition of compound **1** (half maximal inhibitory concentration (IC_50_) value = 11.47 ± 1.57 μM) was equal to that of L-N(6)-(1-iminoethyl)-lysine (IC_50_ = 10.29 ± 1.10 μM) as a positive control.

**Conclusions:**

Some compounds exhibited inhibitory activities against NO production, and the study of structure–activity relationship suggested that uncyclized compounds with oxygen substitution at C-12/12′ showed strong inhibitory activities, and carbonyl units contributed to enhanced activities.

**Supplementary Information:**

The online version contains supplementary material available at 10.1186/s13020-021-00499-y.

## Background

The higher plant, *Psoralea corylifolia* L. (*Cullen corylifolia* (L) Mefik) is an annual herb and belongs to family Leguminosae, distributed in China, India, Malay peninsula, and Indonesia [1]. Dried fruits of *P. corylifolia* (*Psoraleae Fructus*) is one of the most popular traditional Chinese medicine (TCM) and officially listed in Chinese Pharmacopoeia [2], and it is also a natural food additive [3]. It has been used for the treatment of nephritis, spermatorrhea, pollakiuria, asthma, and various inflammatory diseases [4]. *Psoraleae Fructus* contains approximately 110 compounds including coumarins, flavonoids, meroterpenoids, and benzofurans [5]. Among these, meroterpenoids are considered to be the one of characteristic and active components [6, 7].

Bakuchiol is a main meroterpenoid that consists of a side chain (3-ethenyl-3,7-dimethyl-1,6-octadienyl) and a *p*-disubstituted benzene ring. Structural changes including oxidation, dehydration reduction, condensation and alkylation, occur in the side chain and benzene ring, which increases structural and bioactive diversities of meroterpenoid constituents. Remarkably, bakuchiol and its' derivants exhibited extensive bioactivities, such as anti-inflammatory, anti-oxidant, antitumor, antidepressant, antidiabetic and osteoblastic activities [8]. Therefore, people have been trying to find monoterpenes with various biological activities. According to predecessors' researches, 22 meroterpenoids and 12 bakuchiol dimers were found from the plant [5, 9–11]. In our previous researches [12, 13], fourteen meroterpenoids and seventeen heterodimers of bakuchiol have been reported and their anticancer cytotoxicity were evaluated. Further investigation on the cyclohexane extract brought about twelve unpresented bakuchiol dimmers, bisbakuchiols M–U (**1**–**9**) and bisbakuchiol ethers A–C (**10**–**12**), along with five known compounds (**13**–**17**), whose anti-inflammatory activities were evaluated. Herein structure elucidation of these compounds and evaluation of their ability to inhibit nitric oxide (NO) production in lipopolysaccharide (LPS)-stimulated RAW264.7 macrophages were discussed.

## Materials and methods

### General experimental procedures

Infrared data were recorded on a Thermo Nicolet Nexus 470 FT-IR spectrometer. Ultraviolet data were acquired on a Mapada UV-6100 double beam spectrophotometer. HRESIMS data were collected using a Waters Xevo G2 QTOF spectrometer. NMR spectra were recorded on a Bruker AVANCE III HD 400 NMR spectrometer. Optical rotations were measured on a Rudolph Autopol IV automatic polarimeter. X-ray data were collected by a Rigaku Micromax-003 X-ray single-crystal diffractometer with CuKα radiation. Open column chromatography (CC) was performed by packing silica gel (200–300 mesh, Marine Chemical Ltd., Qingdao, China), Sephadex LH-20 gel (Pharmacia Biotek, Denmark). Thin layer chromatography (TLC) was carried out on silica gel GF254 plates (Merck, Darmstadt, Germany) with 10% H_2_SO_4_ in 95% ethanol followed by heating. Reversed phase semi-preparative HPLC (RP-SP-HPLC) was accomplished using an LC3000 system (Beijing Innovation Technology Co., Ltd), equipped with a phenomenon C_18_ column (21.2 mm × 250 mm, 5 μm). Cells were cultured in Sanyo MCO-15 AC carbon dioxide (CO_2_) incubator (Sanyo Electric Co., Ltd., Osaka, Japan).

HPLC grade solvents, methanol (MeOH) and acetonitrile (MeCN), were purchased from Fisher Scientific (Pittsburgh, PA, USA) and solvents, petroleum ether (PE), cyclohexane (cHE), ethyl acetate (EtOAc), chloroform (CHCl_3_) and normal-butanol (BuOH) for column chromatography purchased from Beijing Chemical Works (Beijing, China). Dulbecco's modified Eagle's medium (DMEM), fetal bovine serum (FBS), trypsin, penicillin–streptomycin solution, phosphate buffered saline were obtained from Gibco® (Life Technologies Inc., Grand Island, NY, USA). 3-(4,5-Dimethyl-2-thiazolyl)-2,5-diphenyl-2H-tetrazolium bromide (MTT), lipopolysaccharide (LPS), Griess reagent, dimethylsulfoxide (DMSO), and L-N(6)-(1-iminoethyl)-lysine (L-NIL) were obtained from Sigma-Aldrich (St. Louis, MO, USA). The murine macrophage cell line RAW264.7 was obtained from the Cell Bank of the Chinese Academy of Sciences (Shanghai, China).

### Plant material

The mature fruits of *Psoralea corylifolia* L. were harvested from Yunnan province of People's Republic of China (GPS coordinates:23°32′N, 99°23′E) in October 2016, and authenticated by Prof. Xiu-Wei Yang of the School of Pharmaceutical Sciences, Peking University. A voucher specimen (accession number: BGZ201610) of the fruits was deposited at the State Key Laboratory of Natural Medicines and Biomimetic Drugs of Peking University.

### Extraction and isolation

The dried mature fruits powder (47.9 kg) was extracted with 70% aqueous ethanol under reflux. After extracted for three times (first 479 kg for 2 h, and then 384 kg for 2 h two times), the crude extract (8.2 kg, yield 17.12%) was obtained. And then, part of the residue (6.0 kg) was suspended in H_2_O (8 L) and extracted with cHE (8 L × 8), EtOAc (8 L × 8) and *n*-butanol (BuOH, 8 L × 8) successively and afforded corresponding extract for 1.2 kg, 2.2 kg and 0.7 kg. The cHE extract (1.0 kg) was fractionated by silica gel column (SGC, 140 mm i.d. × 800 mm) with gradient eluent (PE-EtOAc, 5:1, 3:1, 1:1, 2:3, 1:3, 0:1, *v/v*) to give 26 fractions (Fr. A–Z). The Fr. B (27.2 g) was separated by SGC (55 mm i.d. × 650 mm) with a step gradient eluent of PE-EtOAc (100: 1, 50: 1, 25: 1, 7: 1, 5: 1, 3:1, 1: 1, 1:3, 0:1, *v/v*) to afford 15 subfractions (Fr. B-1–Fr. B-15). Fr. B-8 (2.1 g) was separated by reversed-phase column (RPC), eluted with MeCN-H_2_O (40:60 to 100:0, *v/v*), to give 13 subfractions. Fr. B-8–5 was purified by SP-RP-HPLC (MeCN-H_2_O, 95:5, *v/v*), to yield compound **10** (5 mg, *t*_*R*_ = 85 min). The Fr. C (136 g) was separated by SGC (120 mm i.d. × 600 mm) with a step gradient eluent of PE-EtOAc (100: 1, 50: 1, 20: 1, 15: 1, 10: 1, 5:1, 5: 2, 1:1, 0:1, *v/v*) to afford 16 subfractions (Fr. C-1–Fr. C-16). Fr. C-4–7 was separated by Sephadex LH-20 column (SC) and purified by SP-RP-HPLC (MeCN-H_2_O, 93:7, *v/v*), to yield compound **1** (230 mg, *t*_*R*_ = 45 min) and **11** (60 mg, *t*_*R*_ = 80 min). Fr. C-5 (20 g) was separated by RPC, eluted with MeOH-H_2_O (80:20 to 100:0, *v/v*), to give 9 subfractions. Fr.C-5–9 was separated by SC and purified by SP-RP-HPLC (MeCN-H_2_O, 93:7, *v/v*), to yield compound **12** (34 mg, *t*_*R*_ = 71 min). By SP-RP-HPLC (MeCN-H_2_O, 90:10, *v/v*) and preparative TLC (PE-CHCl_3_, 10:1, *v/v*), compound **2** (104 mg, *t*_*R*_ = 252 min) was obtained from Fr. C-11 (2.1 g). The Fr. D (335 g) was separated by SGC (140 mm i.d. × 800 mm) with a step gradient eluent of PE-CHCl_3_ (100: 1, 50: 1, 20: 1, 10: 1, 5: 1, 4:1, 3: 1, 2: 1, 1: 1, 1:3, 0:100, *v/v*) to afford 14 subfractions (Fr. D-1–Fr. D-14). Fr. D-4 (20.7 g) was separated by RPC, eluted with MeOH-H_2_O (45:55 to 100:0, *v/v*), to give 13 subfractions. Fr. D-4–12 was purified by SP-RP-HPLC (MeOH-H_2_O, 93:7, *v/v*), to yield compounds **8** (15 mg, *t*_*R*_ = 131 min) and **9** (28 mg, *t*_*R*_ = 136 min). By SP-RP-HPLC (MeCN-H_2_O, 90:10, *v/v*), Fr. D-7(1.7 g)was separated to yield compounds **3** (5 mg, *t*_*R*_ = 95 min) and **4** (2 mg, *t*_*R*_ = 87 min). The Fr. D-9 (37 g) was separated by SGC (55 mm i.d. × 650 mm) with a step gradient eluent of PE-CHCl_3_ (100: 1, 50: 1, 20: 1, 10: 1, 9: 1, 8:1, 7: 1, 6: 1, 5: 1, 4:1, 3:1, 1:1, 1:3, 0:1, *v/v*) to afford 28 subfractions. By SP-RP-HPLC (MeOH-H_2_O, 92:8, *v/v*), Fr. D-9–13 was separated to yield compounds **5** (11 mg, *t*_*R*_ = 77 min), **6** (9 mg, *t*_*R*_ = 100 min) and **7** (19 mg, *t*_*R*_ = 110 min). Fr. D-9–25 was purified by SP-RP-HPLC (MeOH-H_2_O, 92:8, *v/v*), to give compounds **13** (18 mg, *t*_*R*_ = 86 min) and **14** (25 mg, *t*_*R*_ = 92 min). Fr. D-9–26 was purified by SP-RP-HPLC (MeOH-H_2_O, 92:8, *v/v*), to give compound **15** (15 mg, *t*_*R*_ = 76 min). Fr. D-11 (19.2 g) was separated by RPC, eluted with MeOH-H_2_O (45:55 to 100:0, *v/v*), to give 27 subfractions. The Fr. D-11–25 (5.9 g) was separated by SGC (35 mm i.d. × 500 mm) with a step gradient eluent of PE-CHCl_3_ (8:1, 7: 1, 6: 1, 5: 1, 4:1, 3:1, 1:1, 1:3, 0:100, *v/v*) to afford 18 subfractions. By SP-RP-HPLC (MeOH-H_2_O, 75:25, *v/v*), Fr. D-11–25-12 was separated to yield compounds **16** (14 mg, *t*_*R*_ = 286 min) and **17** (15 mg, *t*_*R*_ = 328 min).

Bisbakuchiol M (**1**). Brown–red needle crystals; mp 114–116 ºC; [*α*]25 D + 50.0 (*c* 0.1, MeOH); UV (MeOH) *λ*_max_ (log *ε*): 204 (4.85), 258 (4.80), 386 (4.28) nm; IR (KBr) *ν*_max_ 3315, 2967, 2930, 1692, 1609, 1582, 1504, 1385, 1358, 1255, 1035 cm^−1^; ^1^H NMR (CDCl_3_, 400 MHz), see Table 1; ^13^C NMR (CDCl_3_, 100 MHz), see Table 2; HRESIMS *m/z* 537.3004 [M + H]^+^ (calcd for C_36_H_41_O_4_, 537.3005).

**Table 1:** ^1^H NMR (400 MHz, CDCl_3_; *δ*_H_, *J* in Hz) data for compounds 1–9

Position	1	2	3	4	5	6	7	8	9
1									
2	7.42 (d, 8.6)	7.26 (d, 2.4)	7.00 (d, 8.6)	6.96 (d, 8.7)	7.17 (d, 8.4)	7.19 (d, 8.6)	7.21 (d, 8.5)	6.93 (br s)	6.92 (br s)
3	7.04 (d, 8.6)		6.43 (d, 8.6)	6.36 (d, 8.7)	6.72 (d, 8.4)	6.72 (d, 8.6)	6.79 (d, 8.5)		
4									
5	7.04 (d, 8.6)	6.97 (d, 8.5)	6.43 (d, 8.6)	6.36 (d, 8.7)	6.72 (d, 8.4)	6.72 (d, 8.6)	6.79 (d, 8.5)	6.86, overlapped	6.85, overlapped
6	7.42 (d, 8.6)	7.34 (dd, 8.5, 2.4)	7.00 (d, 8.6)	6.96 (d, 8.7)	7.17 (d, 8.4)	7.19 (d, 8.6)	7.21 (d, 8.5)	6.85, overlapped	6.85, overlapped
7	6.33 (d, 16.2)	6.29 (d, 16.3)	6.13 (d,16.3)	6.11 (d, 16.2)	6.18 (d, 16.2)	6.25 (d, 16.2)	6.26 (d, 16.2)	6.19 (d, 16.2)	6.19 (d, 16.2)
8	6.20 (d, 16.2)	6.11 (d, 16.3)	5.95 (d, 16.3)	5.93 (d, 16.2)	5.99 (d, 16.2)	6.08 (d, 16.2)	6.09 (d, 16.2)	6.03 (d, 16.2)	6.03 (d, 16.2)
9									
10	1.53 (m)	1.50 (m)	1.45 (m)	1.45 (m)	1.46 (m)	1.49 (m)	1.50 (m)	1.46 (m)	1.46 (m)
11	1.98 (m)	1.96 (m)	1.92 (m)	1.91 (m)	1.93 (m)	1.94 (m)	1.95 (m)	1.93 (m)	1.93 (m)
12	5.12 (m)	5.10 (m)	5.09 (br t, 7.1)	5.08 (br t, 6.9)	5.08 (br t, 7.3)	5.10 (br t, 7.3)	5.10 (br t, 7.3)	5.09 (br t)	5.09 (br t)
13									
14	1.60 (s)	1.58 (s)	1.57 (s)	1.66 (s)	1.56 (s)	1.58 (s)	1.58 (s)	1.57 (s)	1.57 (s)
15	1.68 (s)	1.67 (s)	1.66 (s)	1.56 (s)	1.66 (s)	1.67 (s)	1.67 (s)	1.67 (s)	1.67 (s)
16	1.23 (s)	1.20 (s)	1.15 (s)	1.14 (s)	1.15 (s)	1.19 (s)	1.19 (s)	1.17 (s)	1.17 (s)
17	5.87 (dd, 17.6, 11.0)	5.88 (dd, 17.4, 10.7)	5.84 (dd, 17.2, 10.8)	5.83 (dd, 17.2, 10.8)	5.84 (dd, 17.7, 10.7)	5.87 (dd, 17.5, 10.9)	5.87 (dd, 17.5, 10.8)	5.85 (dd, 17.6, 10.7)	5.85 (dd, 17.6, 10.7)
18a	5.04 (dd, 17.6, 1.3)	5.02 (dd, 17.4, 1.4)	4.97 (dd, 17.2, 1.4)	4.97 (dd, 17.2, 1.4)	4.96 (dd, 17.7, 1.3)	5.01 (dd, 17.5, 1.3)	5.01 (dd, 17.5, 1.3)	5.01 (dd, 10.7, 1.3)	5.01 (dd, 10.7, 1.3)
18b	5.08 (dd, 11.0, 1.3)	5.04 (dd, 10.7, 1.4)	5.00 (dd, 10.8, 1.4)	5.00 (dd, 10.8, 1.4)	5.00 (dd, 10.7, 1.3)	5.03 (dd, 10.9, 1.3)	5.02 (dd, 10.8, 1.3)	4.99 (dd, 17.6, 1.3)	4.99 (dd, 17.6, 1.3)
1′									
2′		7.26 (d, 2.4)	7.00 (d, 8.6)	7.01 (d, 8.7)	7.16 (d, 8.7)	7.24 (d, 8.5)	7.18 (d, 8.4)	7.19 (br d, 8.6)	7.16 (br d, 8.6)
3′	5.68 (s)		6.56 (d, 8.6)	6.56 (d, 8.7)	6.71 (d, 8.7)	6.77 (d, 8.5)	6.74 (d, 8.4)	6.73 (br d, 8.6)	6.77 (br d, 8.6)
4′									
5′		6.97 (d, 8.5)	6.56 (d, 8.6)	6.56 (d, 8.7)	6.71 (d, 8.7)	6.77 (d, 8.5)	6.74 (d, 8.4)	6.73 (br d, 8.6)	6.77 (br d, 8.6)
6′		7.34 (dd, 8.5, 2.4)	7.00 (d, 8.6)	7.01 (d, 8.7)	7.16 (d, 8.7)	7.24 (d, 8.5)	7.18 (d, 8.4)	7.19 (br d, 8.6)	7.16 (br d, 8.6)
7′	7.43 (s)	6.29 (d, 16.3)	2.89 (br dd, 11.7, 10.6)	2.96 (br dd, 11.7, 10.5)	5.17 (d, 2.2)	4.18 (d, 8.7)	4.30 (d, 3.0)	4.80 (d, 7.3)	4.91 (d, 6.1)
8′		6.11 (d, 16.3)	4.06 (d, 10.6)	4.05 (d, 10.5)	3.46 (d, 2.2)	3.96 (d, 8.7)	3.54 (d, 3.0)	3.97 (d, 7.3)	4.04 (d, 6.1)
9′									
10′a	2.60 (d, 18.7)	1.50 (m)	1.98 (m)	1.72 (m)	1.86 (m)	1.76 (m)	2.32 (m)	1.76 (m)	1.55 (m)
10′b	2.46(d, 18.7)		1.56 (m)	1.64 (m)	1.62 (m)	1.76 (m)	1.49 (m)	1.30 (m)	1.44 (m)
11′a		1.96 (m)	1.79 (dd, 12.3, 3.3),	1.70 (dd, 11.8, 3.5)	1.93 (m)	1.74 (m)	1.85 (m)	1.93(m)	1.83 (m)
11′b		1.96 (m)	1.55 (dd, 12.3, 3.3)	1.60 (dd, 11.8, 3.5)	1.88 (m)	1.74 (m)	1.85 (m)	1.82 (m)	1.83 (m)
12′		5.10 (m)	2.53 (dt, 12.3, 3.3)	2.47 (dt, 11.8, 3.5)	4.58 (m)	3.14 (m)	4.08 (dd, 3.0)	5.06 (m)	5.03 (m)
13′									
14′	1.78 (s)	1.58 (s)	4.57 (br s), 4.54 (br s)	4.58 (br s) 4.56 (br s)	4.95 (d, 1.3) 4.91 (d, 1.3)	0.82 (s)	1.11 (s)	1.58 (s)	1.57 (s)
15′	1.78 (s)	1.67 (s)	1.51 (s)	1.56 (s)	1.35 (s)	0.49 (s)	1.09 (s)	1.66 (s)	1.67 (s)
16′	1.44 (s)	1.20 (s)	1.04 (s)	1.30 (s)	1.10 (s)	1.10 (s)	1.17 (s)	0.62 (s)	1.07 (s)
17′	5.87 (dd, 10.6, 7.5)	5.88 (dd, 17.4, 10.7)	6.43 (dd, 17.2, 10.8)	5.83 (dd, 17.2, 10.8)	6.46 (dd, 17.7, 10.9)	6.15 (dd, 17.5, 10.9)	6.02 (dd, 17.5, 10.8)	5.84 (dd, 17.4, 10.8)	5.48 (dd, 17.7, 11.0)
18′a	5.19 (br d, 3.9)	5.02 (dd, 17.4, 1.4)	5.20 (dd, 17.2, 1.4)	5.20 (dd, 17.2, 1.4)	4.81 (d, 1.6)	5.14 (dd, 17.5, 1.3)	5.05 (dd, 17.5, 1.3)		
18′b	5.16 (br d, 10.6)	5.04 (dd, 10.7, 1.4)	5.29 (dd, 10.8, 1.4)	5.29 (dd, 10.8, 1.4)	4.85 (d, 1.6)	4.99 (dd, 10.9, 1.3)	5.02 (dd, 10.8, 1.3)		
OCH3						3.04 (s)	3.16 (s)

**Table 2:** ^13^C NMR (100 MHz, CDCl_3_) data for compounds 1–9

Position	1	2	3	4	5	6	7	8	9
1	136.2, C	131.6, C	130.3, C	129.9, C	130.5, C	132.7, C	132.8, C	131.3, C	131.4, C
2	127.8, CH	123.4, CH	126.6, CH	126.4, CH	126.9, CH	126.3, CH	126.4, CH	113.7, CH	113.9, CH
3	121.4, CH	128.9, C	115.9, CH	116.3, CH	115.2, CH	123.7, CH	124.0, CH	143.7, C	143.4, C
4	152.0, C	152.1, C	159.7, C	159.4, C	157.0, C	155.3, C	154.3, C	143.0, C	143.0, C
5	121.4, CH	116.7, CH	115.9, CH	116.3, CH	115.2, CH	123.7, CH	124.0, CH	116.5, CH	116.4, CH
6	127.8, CH	127.6, CH	126.6, CH	126.4, CH	126.9, CH	126.3, CH	126.4, CH	119.5, CH	119.5, CH
7	125.9, CH	126.1, CH	126.6, CH	126.7, CH	126.5, CH	126.6, CH	126.7, CH	126.5, CH	126.6, CH
8	138.9, CH	136.7, CH	135.3, CH	135.1, CH	135.7, CH	136.5, CH	136.5, CH	136.1, CH	136.1, CH
9	42.7, C	42.6, C	42.4, C	42.4, C	42.5, C	42.5, C	42.5, C	42.5, C	42.5, C
10	41.2, CH_2_	41.2, CH_2_	41.3, CH_2_	41.3, CH_2_	41.2, CH_2_	41.2, CH_2_	41.2, CH_2_	41.3, CH_2_	41.3, CH_2_
11	23.2, CH_2_	23.2, CH_2_	23.3, CH_2_	23.2, CH_2_	23.5, CH_2_	23.2, CH_2_	23.2, CH_2_	23.2, CH_2_	23.2, CH_2_
12	124.6, CH	124.7, CH	124.8, CH	124.8, CH	124.8, CH	124.8, CH	124.8, CH	124.8, CH	124.8, CH
13	131.4, C	131.3, C	131.2, C	131.2, C	131.2, C	131.3, C	131.3, C	131.5, C	131.4, C
14	17.7, CH_3_	17.6, CH_3_	17.6, CH_3_	17.6, CH_3_	17.6, CH_3_	17.6, CH_3_	17.6, CH_3_	17.6, CH_3_	17.6, CH_3_
15	25.7, CH_3_	25.7, CH_3_	25.7, CH_3_	25.7, CH_3_	25.7, CH_3_	25.7, CH_3_	25.7, CH_3_	25.7, CH_3_	25.7, CH_3_
16	23.2, CH_3_	23.3, CH_3_	23.3, CH_3_	23.3, CH_3_	23.4, CH_3_	23.3, CH_3_	23.3, CH_3_	23.4, CH_3_	23.4, CH_3_
17	145.5, CH	145.8, CH	146.0, CH	146.0, CH	146.0, CH	145.9, CH	145.9, CH	145.9, CH	145.9, CH
18	112.3, CH_2_	112.0, CH_2_	111.7, CH_2_	111.7, CH_2_	111.8, CH_2_	111.9, CH_2_	111.9, CH_2_	113.9, CH_2_	111.8, CH_2_
1′	131.6, C	131.6, C	133.3, C	133.3, C	131.9, C	131.5, C	132.4, C	130.4, C	130.4, C
2′	163.8, C	123.4, CH	126.6, CH	129.9, CH	128.0, CH	130.6, CH	128.9, CH	129.8, CH	129.6, CH
3′	104.5, CH	128.9, C	114.7, CH	114.7, CH	115.7, CH	114.8, CH	114.8, CH	115.4, CH	115.2, CH
4′	180.4, C	152.1, C	153.6, C	153.6, C	154.8, C	154.1, C	154.7, C	156.1, C	156.0, C
5′	144.8, C	116.7, CH	114.7, CH	114.7, CH	115.7, CH	114.8, CH	114.8, CH	115.4, CH	115.2, CH
6′	146.8, C	127.6, CH	126.6, CH	129.9, CH	128.0, CH	130.6, CH	128.9, CH	129.8, CH	129.6, CH
7′	126.6, CH	126.1, CH	49.0, CH	48.1, CH	79.9, CH	82.9, CH	84.8, CH	77.4, CH	76.4, CH
8′	122.7, C	136.7, CH	89.1, CH	87.8, CH	83.1, CH	80.5, CH	83.8, CH	81.8, CH	81.4, CH
9′	43.5, C	42.6, C	43.5, C	42.5, C	39.0, C	38.6, C	39.2, C	43.0, C	43.6, C
10′	51.4, CH_2_	41.2, CH_2_	36.6, CH_2_	35.5, CH_2_	34.0, CH_2_	32.8, CH_2_	30.7, CH_2_	38.3, CH_2_	38.8, CH_2_
11′	204.2, C	23.2, CH_2_	27.9, CH_2_	27.2, CH_2_	23.2, CH_2_	20.5, CH_2_	20.3, CH_2_	22.2, CH_2_	22.2, CH_2_
12′	163.0, C	124.7, CH	51.0, CH	50.7, CH	76.0, CH	76.3, CH	78.4, CH	124.5, CH	124.6, CH
13′	37.7, C	131.3, C	147.0, C	146.9, C	144.6, C	82.0, C	82.7, C	131.6, C	131.3, C
14′	22.3, CH_3_	17.6, CH	111.9, CH_2_	112.0, CH_2_	111.5, CH_2_	20.9, CH_3_	24.2, CH_3_	17.5, CH_3_	17.5, CH_3_
15′	22.5, CH_3_	25.7, CH_3_	19.2, CH_3_	19.3, CH_3_	19.3, CH_3_	23.4, CH_3_	21.9, CH_3_	25.7, CH_3_	25.7, CH_3_
16′	23.9, CH_3_	23.3, CH_3_	28.7, CH_3_	17.1, CH_3_	24.3, CH_3_	24.0, CH_3_	23.3, CH_3_	19.9, CH_3_	17.4, CH_3_
17′	142.5, CH	145.8, CH	141.2, CH	147.5, CH	143.4, CH	146.7, CH	145.8, CH	141.9, CH	141.9, CH
18′	114.5, CH_2_	112.0, CH_2_	114.2, CH_2_	111.6, CH_2_	112.2, CH_2_	111.7, CH_2_	111.1, CH_2_	111.8, CH_2_	112.4, CH_2_
OCH3						55.1	56.8		

Bisbakuchiol N (**2**). Yellow oils; [*α*]25 D + 20.0 (*c* 0.1, MeOH); UV (MeOH) *λ*_max_ (log *ε*): 203 (4.41), 253 (4.44) nm; IR (KBr) *ν*_max_ 3319, 2966, 2924, 1703, 1633, 1603, 1496, 1409, 1373, 1231, 969 cm^−1^; ^1^H NMR (CDCl_3_, 400 MHz), see Table 1; ^13^C NMR (CDCl_3_, 100 MHz), see Table 2; HRESIMS *m/z* 511.3573 [M + H]^+^ (calcd for C_36_H_47_O_2_, 511.3576).

Bisbakuchiol O (**3**). Yellowish oils; [*α*]25 D + 30.0 (*c* 0.1, MeOH); UV (MeOH) *λ*_max_ (log *ε*): 203 (4.57), 267 (4.33) nm; IR (KBr) *ν*_max_ 3373, 2962, 2926, 1704, 1604, 1507, 1454, 1372, 1238, 1172, 1007 cm^−1^; ^1^H NMR (CDCl_3_, 400 MHz), see Table 1; ^13^C NMR (CDCl_3_, 100 MHz), see Table 2; HRESIMS *m/z* 555.3468 [M + HCOO]^−^ (calcd for C_37_H_47_O_4_, 555.3474).

Bisbakuchiol P (**4**). Yellowish oils; [*α*]25 D − 26.7 (*c* 0.1, MeOH); UV (MeOH) *λ*_max_ (log *ε*): 202 (4.61), 267 (4.32) nm; IR (KBr) *ν*_max_ 3381, 2968, 2927, 1703, 1604, 1507, 1452, 1375, 1240, 1172, 1000 cm^−1^; ^1^H NMR (CDCl_3_, 400 MHz), see Table 1; ^13^C NMR (CDCl_3_, 100 MHz), see Table 2; HRESIMS *m/z* 509.3417 [M – H]^−^ (calcd for C_36_H_45_O_2_, 509.3420).

Bisbakuchiol Q (**5**). Yellowish oils; [*α*]25 D + 70.0 (*c* 0.1, MeOH); UV (MeOH) *λ*_max_ (log *ε*): 202 (4.76), 264 (4.55) nm; IR (KBr) *ν*_max_ 3370, 2964, 2921, 1704, 1607, 1507, 1459, 1370, 1238, 1171, 1099 cm^−1^; ^1^H NMR (CDCl_3_, 400 MHz), see Table 1; ^13^C NMR (CDCl_3_, 100 MHz), see Table 2; HRESIMS *m/z* 525.3364 [M – H]^−^ (calcd for C_36_H_45_O_3_, 525.3369).

Bisbakuchiol R (**6**). White amorphous powder; [*α*]25 D + 70.0 (*c* 0.1, MeOH); UV (MeOH) *λ*_max_ (log *ε*): 203 (4.57), 260 (4.35) nm; IR (KBr) *ν*_max_ 3372, 2967, 2924, 1704, 1613, 1506, 1451, 1365, 1253, 1143, 1094 cm^−1^; ^1^H NMR (CDCl_3_, 400 MHz), see Table 1; ^13^C NMR (CDCl_3_, 100 MHz), see Table 2; HRESIMS *m/z* 603.3676 [M + HCOO]^−^ (calcd for C_38_H_51_O_6_, 603.3686).

Bisbakuchiol S (**7**). White amorphous powders; [*α*]25 D + 60.0 (*c* 0.1, MeOH); UV (MeOH) *λ*_max_ (log *ε*): 204 (4.42), 260 (4.32) nm; IR (KBr) *ν*_max_ 3373, 2968, 2925, 1705, 1614, 1506, 1450, 1364, 1235, 1143, 1082 cm^−1^; ^1^H NMR (CDCl_3_, 400 MHz), see Table 1; ^13^C NMR (CDCl_3_, 100 MHz), see Table 2; HRESIMS *m/z* 557.3635 [M − H]^−^ (calcd for C_37_H_49_O_4_, 557.3631).

Bisbakuchiol T (**8**). Yellowish oils; [*α*]25 D − 20.0 (*c* 0.1, MeOH); UV (MeOH) *λ*_max_ (log *ε*): 202 (4.68), 222 (4.57), 262 (4.33) nm; IR (KBr) *ν*_max_ 3395, 2967, 2921, 1702, 1588, 1507, 1450, 1375, 1267, 1171, 1010 cm^−1^; ^1^H NMR (CDCl_3_, 400 MHz), see Table 1; ^13^C NMR (CDCl_3_, 100 MHz), see Table 2; HRESIMS *m/z* 525.3367 [M − H]^−^ (calcd for C_36_H_45_O_3_, 525.3369).

Bisbakuchiol U (**9**). Yellowish oils; [*α*]25 D + 20.0 (*c* 0.1, MeOH); UV (MeOH) *λ*_max_ (log *ε*): 202 (4.63), 265 (4.21) nm; IR (KBr) *ν*_max_ 3387, 2966, 2922, 1703, 1587, 1507, 1451, 1374, 1267, 1171, 1009 cm^−1^; ^1^H NMR (CDCl_3_, 400 MHz), see Table 1; ^13^C NMR (CDCl_3_, 100 MHz), see Table 2; HRESIMS *m/z* 525.3371 [M − H]^−^ (calcd for C_36_H_45_O_3_, 525.3369).

Bakuchiol ether A (**10**)*.* Yellowish oils; [*α*]25 D + 10.0 (*c* 0.1, MeOH); UV (MeOH) *λ*_max_ (log *ε*): 204(4.26),260(4.15) nm; IR (KBr) *ν*_max_ 3424, 2969, 2929, 1712, 1603, 1505, 1453, 1369, 1224, 1136, 913 cm^−1^; ^1^H NMR (CDCl_3_, 400 MHz), see Table 3; ^13^C NMR (CDCl_3_, 100 MHz), see Table 3; HRESIMS *m/z* 445.3080 [M + Na]^+^ (calcd for C_29_H_42_O_2_Na, 445.3083).

**Table 3:** ^1^H NMR (400 MHz, CDCl_3_; *δ*_H_, *J* in Hz) data and ^13^C NMR (100 MHz, CDCl_3_) data for compounds **10**–**12**

Position	**10**		**11**		**12**	
	*δ*_H_ (*J* in Hz)	*δ*_C_	*δ*_H_ (*J* in Hz)	*δ*_C_	*δ*_H_ (*J* in Hz)	*δ*_C_
1		134.9, C		130.3, C		131.4, C
2	7.27 (d, 8.6)	126.6, CH	7.25 (d, 8.7)	127.1, CH	7.20 (d, 8.7)	126.4, CH
3	6.93 (d, 8.6)	124.5, CH	6.82 (d, 8.7)	115.9, CH	6.79 (d, 8.7)	121.6, CH
4		153.5, C		158.2, C		154.7, C
5	6.93 (d, 8.6)	124.5, CH	6.82 (d, 8.7)	115.9, CH	6.79 (d, 8.7)	121.6, CH
6	7.27 (d, 8.6)	126.6, CH	7.25 (d, 8.7)	127.1, CH	7.20 (d, 8.7)	126.4, CH
7	6.28 (d, 16.3)	126.5, CH	6.25 (d, 16.2)	126.6, CH	6.25 (d, 16.3)	126.7, CH
8	6.12 (d, 16.3)	137.1, CH	6.04 (d, 16.2)	135.5, CH	6.06 (d, 16.3)	136.0, CH
9		42.6, C		42.5, C		42.5, C
10	1.49 (m)	41.2, CH_2_	1.49 (m)	41.3, CH_2_	1.49 (m)	41.3, CH_2_
11	1.94 (m)	23.2, CH_2_	1.95 (m)	23.2, CH_2_	1.95 (m)	23.2, CH_2_
12	5.12 (m)	124.8, CH	5.11 (br t, 7.1)	124.8, CH	5.11 (m)	124.8, CH
13		131.3, C		131.2, C		131.3, C
14	1.58 (s)	17.7, CH_3_	1.57 (s)	17.6, CH_3_	1.58 (s)	17.7, CH_3_
15	1.67 (s)	25.7, CH_3_	1.67 (s)	25.7, CH_3_	1.67 (s)	25.7, CH_3_
16	1.20 (s)	23.3, CH_3_	1.19 (s)	23.4, CH_3_	1.19 (s)	23.4, CH_3_
17	5.88 (dd, 17.4, 10.8)	145.8, CH	5.88 (dd, 17.4, 10.7)	146.1, CH	5.87 (dd, 17.4, 10.7)	146.0, CH
18a	5.10 (dd, 17.4, 1.3)	111.9, CH_2_	5.01 (dd, 17.4, 1.3)	111.8, CH_2_	5.01 (dd, 17.4, 1.3)	111.9, CH_2_
18b	5.04 (dd, 10.8, 1.3)		5.03 (dd, 10.7, 1.3)		5.03 (dd, 10.7, 1.3)	
1′	1.25 (s),	21.8, CH_3_		45.3, C		80.2, C
2′		84.1, C	4.24 (dd, 8.3, 5.4)	86.2, CH	2.35 (ddd, 11.7, 9.1, 8.6')	40.5, CH
3′	1.26 (s)	26.3, CH_3_	1.87 (dd, 12.9, 5.4), 1.65(m)	44.0, CH_2_	1.86 (dd, 9.1, 6.1), 1.72 (dd, 9.1, 8.6)	30.7, CH_2_
4′	2.31 (m)	53.7, CH		38.3, C		34.7, C
5′	4.01 (d, 9.0)	79.8, CH	1.58 (br d, 14.8)	50.1, CH	2.04 (ddd, 11.7, 9.1, 6.1)	44.9, CH
6′		47.8, C	1.39 (m), 1.49 (m)	20.9, CH_2_	1.41 (m), 1.53 (m)	21.3, CH_2_
7′	1.61 (m), 1.52 (m)	35.1, CH_2_	1.38 (m), 1.16 (m)	33.2, CH_2_	1.50 (m), 1.19 (m)	36.1, CH_2_
8′	1.95 (m), 1.49 (m)	22.8, CH_2_		34.7, C		39.2, C
9′	5.95 (dd, 17.6, 10.8)	146.7, CH	3.34 (br t, 2.6)	75.0, CH	3.50 (t, 4.7)	71.8, CH
10′a	5.03 (dd, 10.8, 1.3)	112.0, CH_2_	2.03 (m),	27.2, CH_2_	2.01 (dddd, 15.5, 12.5, 5.5, 3.0)	28.3, CH_2_
10′b	5.02 (dd, 17.6, 1.3)		1.80 (dd, 14.1, 5.1)		1.78 (ddt, 15.5, 5.5, 3.0)	
11′a	1.10 (s)	17.2, CH_3_	1.38 (m)	26.7, CH_2_	1.71 (m)	37.5, CH_2_
11′b			1.06 (m)			
12′			1.56 (br s), 1.02 (br s)	36.0, CH_2_	1.64 (d, 16.4), 1.49 (d, 16.4)	42.1, CH_2_
13′			0.94 (s)	25.7, CH_3_	1.01 (s)	20.9, CH_3_
14′			1.06 (s)	31.4, CH_3_	1.00 (s)	30.3, CH_3_
15′			0.95 (s)	28.4, CH_3_	0.92 (s)	26.4, CH_3_

Bakuchiol ether B (**11**). Yellowish oils; [*α*]25 D + 20.0 (*c* 0.1, MeOH); UV (MeOH) *λ*_max_ (log *ε*): 206(4.50), 265(4.45) nm; IR (KBr) *ν*_max_ 3420, 2926, 2864, 1715, 1606, 1507, 1463, 1364, 1245, 1173, 969 cm^−1^; ^1^H NMR (CDCl_3_, 400 MHz), see Table 3; ^13^C NMR (CDCl_3_, 100 MHz), see Table 3; HRESIMS *m/z* 477.3713 [M + H]^+^ (calcd for C_33_H_49_O_2_, 477.3733).

Bakuchiol ether C (**12**)*.* Yellowish oils; [*α*]25 D + 30.0 (*c* 0.1, MeOH); UV (MeOH) *λ*_max_ (log *ε*): 206(4.46), 263(4.42) nm; IR (KBr) *ν*_max_ 3420, 2952, 2927, 1710, 1604, 1505, 1452, 1375, 1241, 1171, 967 cm^−1^; ^1^H NMR (CDCl_3_, 400 MHz), see Table 3; ^13^C NMR (CDCl_3_, 100 MHz), see Table 3; HRESIMS *m/z* 475.3532 [M − H]^−^ (calcd for C_33_H_47_O_2_, 475.3576).

### X-ray crystallographic analysis

The X-ray crystallographic experiments were carried out on a XtaLAB Synergy R, HyPix diffractometer with *CuKα* radiation. Crystallographic data (No. CCDC 1993852) of **1** have been deposited at the Cambridge Crystallographic Data Center.

Crystallographic data of **1**: C_72_H_80_O_8_, *M* = 1073.36, *a* = 13.2661(2) Å, *b* = 14.3761(2) Å, *c* = 31.3617(5) Å, *α* = 90°, *β* = 90°, *γ* = 90°, *V* = 5981.13(18) Å^3^, *T* = 100 K, space group *P2*_*1*_*2*_*1*_*2*_*1*_, *Z* = 4, *μ* (Cu Kα) = 0.599 mm^−1^, Crystal size = 0.99 × 0.4 × 0.02 mm^3^, 2Ɵ range for data collection = 8.344 to 139.15826790°, 26,790 reflections measured, 10,867 independent reflections (*R*_*int*_ = 0.0431, *R*_*sigma*_ = 0.0446). The final *R*_*1*_ value was 0.0475 (*I* > *2σ(I)*). The final *wR (F*^*2*^*)* value was 0.1153. Flack parameter =  − 0.02 (12).

### ECD calculations

The calculation was performed by the Gaussian 16 software. Conformation analysis were proceeded with the MMFF94s molecular mechanics force field. Optimization of the stable conformers with a Boltzmann distribution over 1% was conducted by time-dependent density functional theory (TD-DFT) at the Cam-B3LYP/6–31 + G(d, p) level for compounds **8** and **9**, with the CPCM model in MeOH. The ECD data was analysed by SpecDis v1.71 with the half-bandwidth no more than 0.3 eV. The final ECD spectra were obtained based on the Boltzmann-calculated contribution of each conformer.

### Inhibition assay on NO production

RAW264.7 cells were maintained in DMEM containing 10% FBS, in a constant humidity atmosphere of 5% CO_2_ and 95% air at 37 °C. The cells were cultivated at a density of 3 × 10^5^ cells/mL for 24 h in 96-well culture plates. And then, the cells were stimulated with LPS (1 μg/mL) and treated with various concentrations (1.56–50.00 μM) of assay compounds. After exposure to the compounds for 24 h, MTT (20 μL, 5 mg/mL) was added to each well [14]. 4 h later, 100 μL of lysis solution (40 g SDS, 20 mL isopropanol, 0.4 mL concentrated HCl and 400 mL ddH_2_O) was added to dissolve the formazan crystals. Absorbances at 490 nm were measured after 10 h by a Multiskan MK3 Automated Microplate Reader (Thermo-Labsystems, Franklin, MA, USA).

The RAW264.7 cells were grown at a density of 3 × 10^5^ cells/mL in 96-well culture plates. After 24 h, the cells were stimulated with LPS (1 μg/mL) and treated with various non-cytotoxic concentrations of assay compounds. And then, the cell culture supernatant (100 μL) was collected and reacted with the same volume of Griess reagent (100 μL) for 15 min at room temperature [15]. The absorbance was determined at 540 nm. The experiments were performed in parallel for three times, and L-NIL was used as a positive control. IC_50_ (half maximal inhibitory concentration) value of each compound was defined as the concentration (*μ*M) that caused 50% inhibition of NO production.

### Statistical analysis

Data were analyzed by SPSS statistics package v.20.0 (SPSS Inc., Chicago, IL, USA). Results were expressed as the mean ± SD. Students't-test was used for Statistical significances calculation, and p < 0.05 was considered to be statistically significant.

## Results

**Fig. 1:**
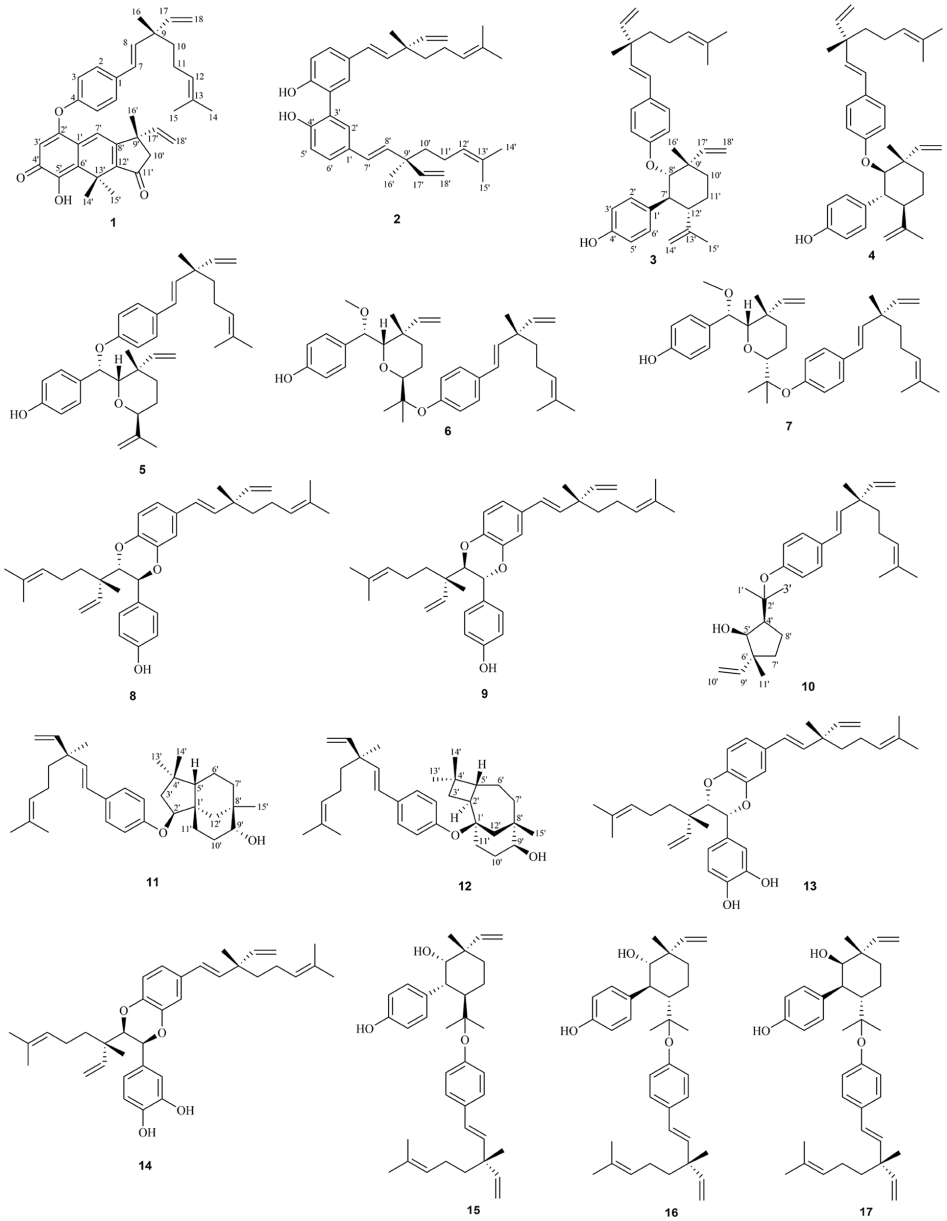
Structures of compounds **1**–**17**

Phytochemical investigation on cHE fraction of 70% ethanol extract of *Psoraleae Fructus* resulted in twelve unpresented bakuchiol dimmers (**1**–**12**) and five known compounds (**13**–**17**) (Fig. 1). Structures of these new compounds were assigned by NMR spectra and single crystal X-ray diffraction. Compounds **1**–**3**, **6**–**9**, and **13**–**17** could be detected from ultrasonic extraction of *Psoraleae Fructus* by LC/MS analysis, suggesting that these compounds were natural products (Additional file 1: Fig. S1).

**Fig. 2:**
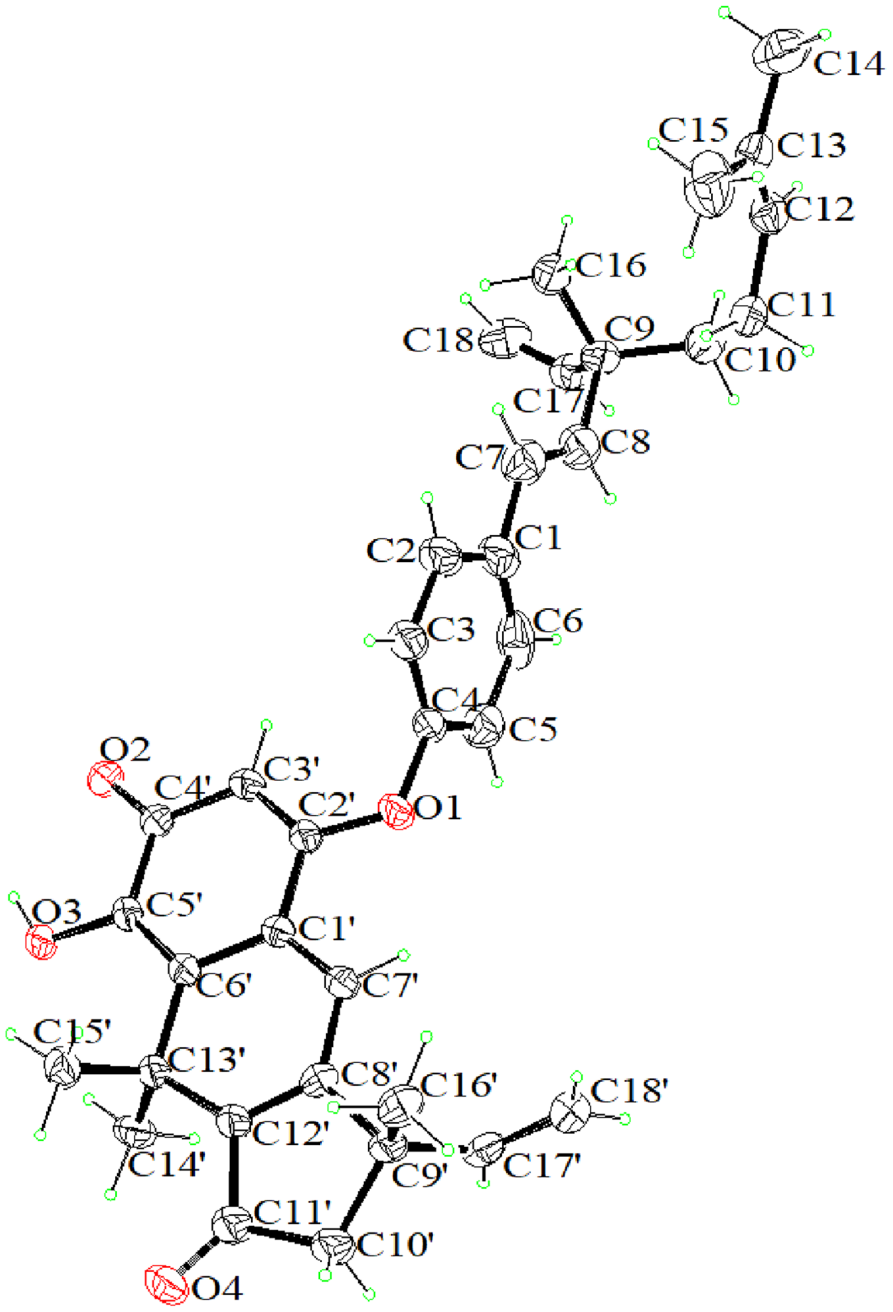
X-ray ORTEP drawings of **1**

Compound **1** was obtained as brown–red needle crystals (MeOH) with mp 114–116 ºC. It had the molecular formula C_36_H_40_O_4_, as established by HRESIMS at *m/z* 537.3004 [M + H]^+^ (calcd for 609.3216). Compared with the NMR data (Tables 1 and 2) of bakuchiol [16], a side chain (3-ethenyl-3,7-dimethyl-1,6-octadienyl) and a *p*-disubstituted benzene ring in **1** were identical to that of bakuchiol. The ^1^H NMR data of an another side chain of compound **1** exhibited three methyl groups at *δ*_H_ 1.78 (3H, s), 1.78 (3H, s) and 1.44 (3H, s); a vinyl group at *δ*_H_ 5.87 (1H, dd, *J* = 10.6, 7.5 Hz, H-17'), 5.19 (1H, br d, J = 3.9 Hz, Ha-18'), and 5.16 (1H, br d, *J* = 10.6, Hb-18'); two trisubstituted olefinic protons at *δ*_H_ 5.68 (1H, s) and 7.43 (1H, s); and one methylene group at *δ*_H_ 2.60 (1H, d, *J* = 18.7 Hz, Ha-10') and 2.46 (1H, d, *J* = 18.7 Hz, Hb-10'). The presence of an α,β-unsaturated ketone group was revealed by the band at 1692 cm^–1^ in its IR spectrum, which was confirmed by the resonance at *δ*_C_ 180.4(s) in its ^13^C NMR spectrum. Comparison of the ^13^C NMR spectrum of **1** with those of bakuchiol, the chemical shifts of C-3 and C-5 were shifted downfield to *δ*_C_ 121.4, suggesting that this substituted group was connected to C-4 of bakuchiol moiety by an ether linkage. The full assignment of ^1^H and ^13^C NMR resonances was supported by ^1^H–^1^H COSY, DEPT, HSQC and HMBC spectral analyses. The X-ray structure was shown in Fig. 2 and confirmed the absolute configuration of 9*S*,9'*S* for **1**. Thus, the structure of **1** was as shown in Fig. 1 and named bisbakuchiol M.

**Fig. 3:**
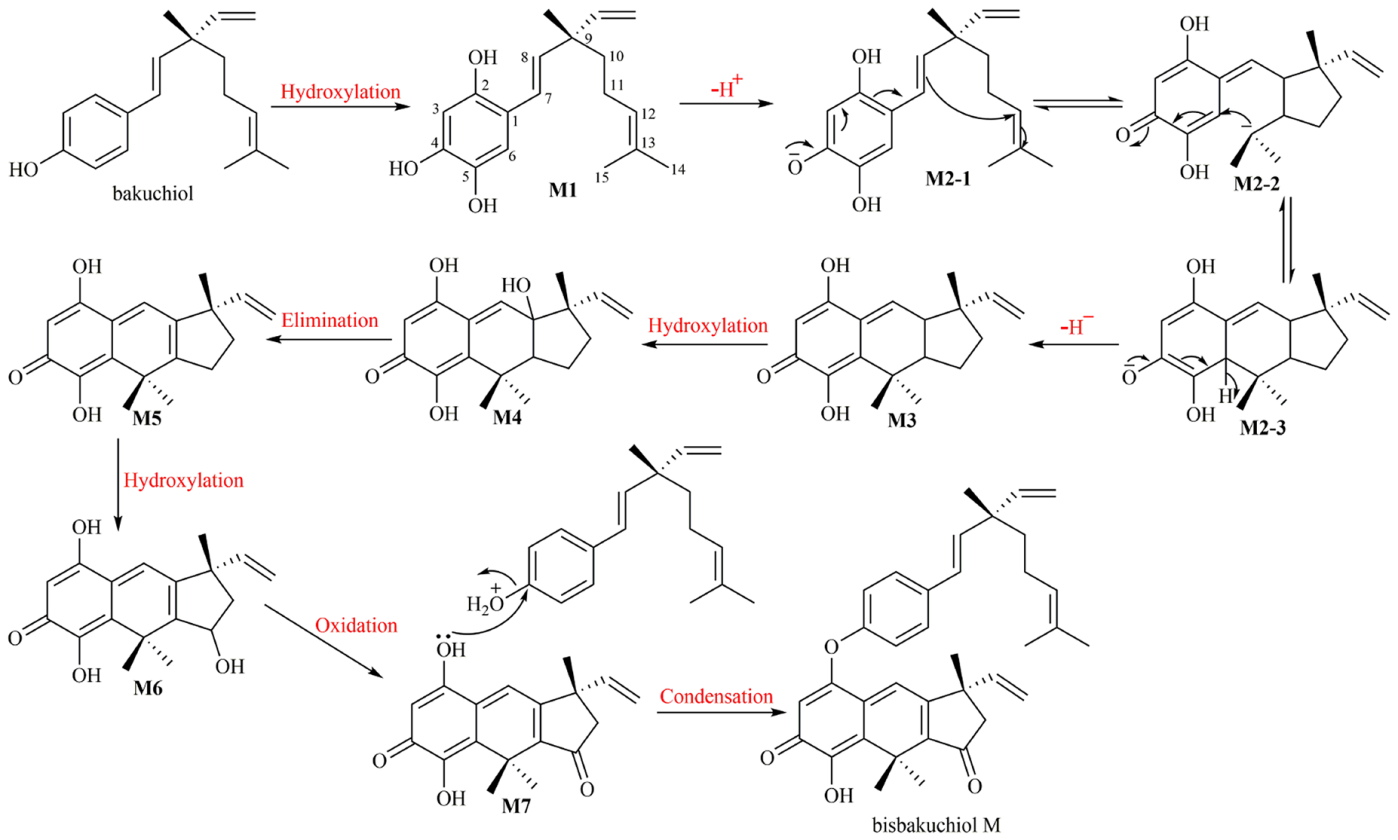
Plausible biogenetic pathway of **1**

The plausible biosynthetic pathway of bisbakuchiol M was proposed (Fig. 3). Hydroxylation reactions occurred at the positions of C-2 and C-5 in bakuchiol to form M1. Once the 4-hydroxyl group in M1 lost a proton to generate M2-1, migrations of the double bond would start. The double bond at C-7 and C-8 would attack C-12 to form a five-membered ring, along with the generation of carbanion at C-13 (M2-2). Subsequently, the carbanion at C-13 attacked C-6 to form six-membered ring (M2-3). The proton at C-6 left, which was accompanied by electron migrations of negative ion of oxygen to produce ketone carbonyl (M3). And then, the α-proton of double bond at C-8 was easily to be hydroxylated to generate M4. The elimination reaction would follow to the generation of M5. Similarly, the hydroxylation occurred at C-11 (M6). Subsequently, 11-hydroxyl group would be oxidized to ketone carbonyl (M7). Finally, M7 and bakuchiol were condensed to produce bisbakuchiol M.

Compound **2** was isolated as yellow oils, had a molecular formula C_36_H_46_O_2_ on the basis of the HRESIMS ion at *m/z* 511.3573 [M + H]^+^. Compared with the NMR data of bakuchiol, a side chain in **2** was identical to that of bakuchiol. The resonances in the ^1^H NMR spectrum [*δ*_H_ 7.26, d, 2.4 Hz; 6.97, d, 8.5 Hz; and 7.34, dd, 8.5, 2.4 Hz] in **2** suggested clearly the 2,5,6-nature of the aromatic protons. Consequently, the structure of compound **2** was unambiguously identified as a dimer comprising two bakuchiol units by a C–C linkage, and it was given the trivial name bisbakuchiol N.

**Fig. 4:**
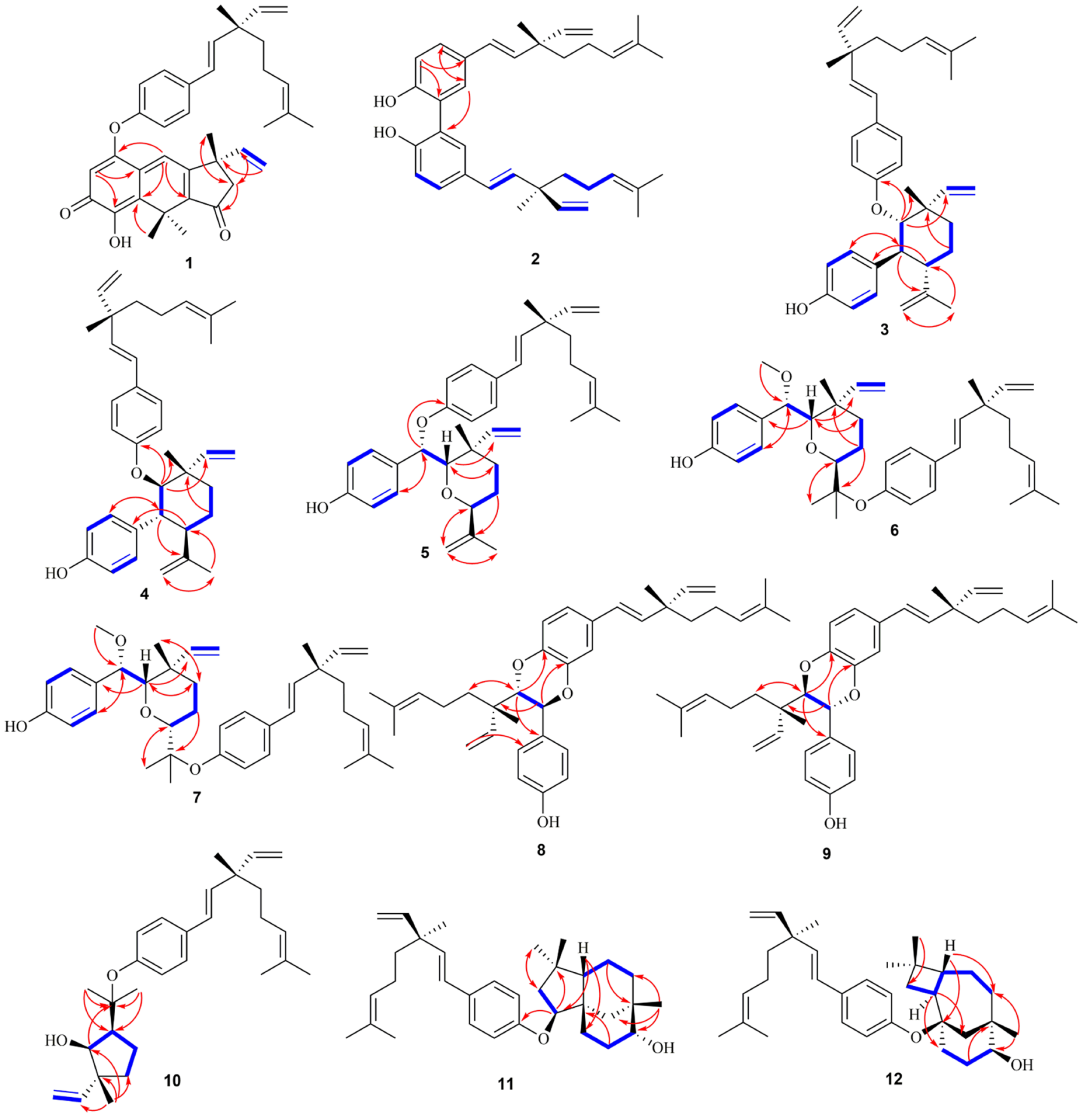
Key ^1^H–^1^H COSY and HMBC correlations of **1**–**12**

Compound **3** was isolated as yellowish oils with [α]25 D + 30.0, and possessed a molecular formula of C_36_H_46_O_2_ by the HRESIMS ion at *m/z* 555.3468 [M + HCOO]^–^. The IR spectrum of **3** showed absorption bands at 3373, 1604, 1507, 1454, 1238, 1007 cm^–1^ ascribable to hydroxyl group and ether functions and aromatic ring. Compared with the NMR data of bakuchiol, a side chain (3-ethenyl-3,7-dimethyl-1,6-octadienyl) and a *p*-disubstituted benzene ring in **3** were identical to that of bakuchiol, together with a set of remaining NMR signals, which were very similar to those of psoracorylifol F characterized from the fruits of *P. corylifolia* [17]. However, the NOE correlation between H-17' at *δ*_H_ 6.43 and H-7' at *δ*_H_ 2.89 was observed, which indicated that H-7' was α-oriented. The large coupling constant (*J* = 11.7 Hz) of H-7' and H-12' indicated a *trans* configuration of the two methine protons. Likewise, the configuration of H-8' was confirmed β-oriented on the basis of the large coupling constant (*J* = 10.6 Hz). Thus, the configuration was assigned as 7'*S*,8'*S*,9'*S*,12'*S* from the occurrence of (9*S*)-bakuchiol only from nature [18, 19]. Furthermore, the HMBC cross-peaks of H-8' at *δ*_H_ 4.06 with aromatic C-4 at *δ*_C_ 159.7 indicated that C-8' was connected to C-4 of bakuchiol moiety by an ether linkage (Fig. 4). According to the above data, the structure of compound **3**, named bisbakuchiol O, was established as shown in Fig. 1.

Compound **4** was also isolated as yellowish oils with [α]25 D–26.7 (*c* 0.1, MeOH), and possessed the same molecular formula, C_36_H_46_O_3_, as **3** according to the HRESIMS *m/z* 509.3417 [M – H]^–^. Similarly, NMR data (Tables 1 and 2) for compound **4** was comparable to those of compound **3**. Compared with compound **3**, H-7' at *δ*_H_ 2.96 (1H, br dd, *J* = 11.7, 10.5 Hz) displayed a NOE correlation with 16'-CH_3_ at *δ*_H_ 1.30 (1H, s), indicating that they were cofacial, and H-7' was assigned in a β-configuration. And the large coupling constants (*J* = 11.7, 10.5 Hz) indicated that H-8' and H-12' were α-oriented. As a result, the configuration was confirmed as 7'*R*,8'*R*,9'*S*,12'*R*. According to the above data, compound **4** was a dimer, whose C-8' of psoracorylifol F was connected to aromatic C-4 of bakuchiol moiety by an ether linkage (Fig. 4). Thus, the structure of compound **4**, named bisbakuchiol P, was established as shown in Fig. 1.

**Fig. 5:**
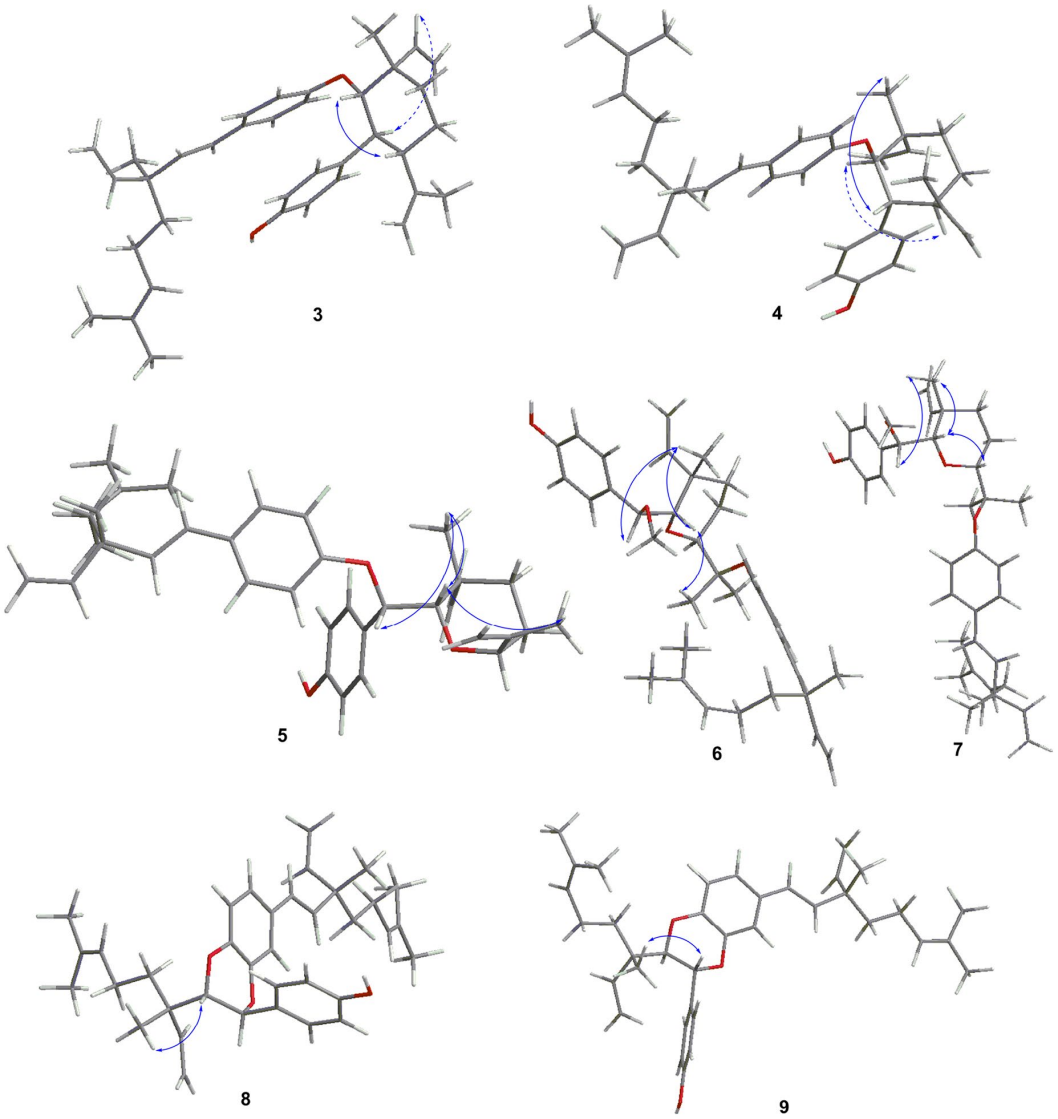
Key NOESY correlations of **3**–**9**

Compound **5** possessed the molecular formula of C_36_H_46_O_3_ as determined by its HRESIMS ion at *m/z* 525.3364 [M–H]^–^. Combined with NMR data, a set of bakuchiol unit signals except for downfield shift to *δ*_C_ 157.0 for C-4, and a set of psoracorylifol A unit signals [20] except for downfield shift to *δ*_C_ 79.9 for C-7' were observed. In the HMBC spectrum of **5** (Fig. 4), a psoracorylifol A unit located at C-4 of the bakuchiol unit was verified by correlations from H-7' at *δ*_H_ 5.17 to C-4 at *δ*_C_ 157. These features permitted assignment of the planar structure of compound **5** as shown in Fig. 1. In the NOESY spectrum (Fig. 5), correlations between H-7' at *δ*_H_ 5.17 and CH_3_-16'β at *δ*_H_ 1.10, H-8' at *δ*_H_ 3.46 and CH_3_-16'β, indicated that H-7' and H-8' were β-oriented. Whereas H-12' at *δ*_H_ 4.58 was α-oriented, which was verified by the NOE correlation from H-8' and CH_3_-15' at *δ*_H_ 1.35. Thus, the absolute structure of compound **5**, named bisbakuchiol Q, was established as 9*S*,7'*S*,8'*S*,9'*S*,12'*S*.

Compound **6** was isolated as white amorphous powders, and possessed the molecular formula of C_37_H_50_O_4_ as deduced by HREIMS *m/z* 603.3676 [M + HCOO]^–^. Similar NMR signals of bakuchiol and psoracorylifol A to **5** were observed in **6**. In the HMBC experiment (Fig. 4), a characteristic methoxyl group at *δ*_H_ 3.04 (3H, s) correlated with C-7' enabled us to attach this methoxyl group to the C-7'. In NMR spectra of **6**, the signals of an *exo*-methylene of psoracorylifol A unit had disappeared, while a new signal for characteristic methyl group at *δ*_H_ 0.49 (3H, s) and an oxygenated quaternary carbon at *δ*_C_ 82.0 had appeared. Combined with the downfield shift of C-3 and C-5 of bakuchiol unit, it was obvious that two units were attached together by C_4_–O–C_13'_. In the NOESY spectrum (Fig. 5), correlations between H-7' at *δ*_H_ 4.18 and CH_3_-16'β at *δ*_H_ 1.10, H-8' at *δ*_H_ 3.96 and CH_3_-16'β, indicated that they were cofacial and were β-oriented. Similarly, the NOE correlation between H-8' and CH_3_-15' at *δ*_H_ 0.82 supported that H-12' at *δ*_H_ 3.14 was α-oriented. Finally, the structural assignment of **6** was assigned as 9*S*,7'*S*,8'*S*,9'*S*,12'*S*, and compound **6** was named bisbakuchiol R.

Compound **7** was isolated as white amorphous powders, and possessed the same molecular formula, C_37_H_50_O_4_, as **6** according to the HRESIMS data (*m/z* 557.3635 [M – H]^–^). Its 1D NMR pattern was highly overlapped to that of compound **6**, indicating their same planar structure. In the NOESY spectrum (Fig. 5), correlations between H-7' at *δ*_H_ 4.30, H-8' at *δ*_H_ 3.54, and CH_3_-16'β at *δ*_H_ 1.17 indicated that they were cofacial and were β-oriented. Meanwhile, the correlation between H-8' and H-12' at *δ*_H_ 4.08 suggested β-orientation of H-12'. Finally, the structural assignment of **7** was 9S,7'*S*,8'*S*,9'*S*,12'*R* as shown in Fig. 1 and compound **7** was named bisbakuchiol S.

**Fig. 6:**
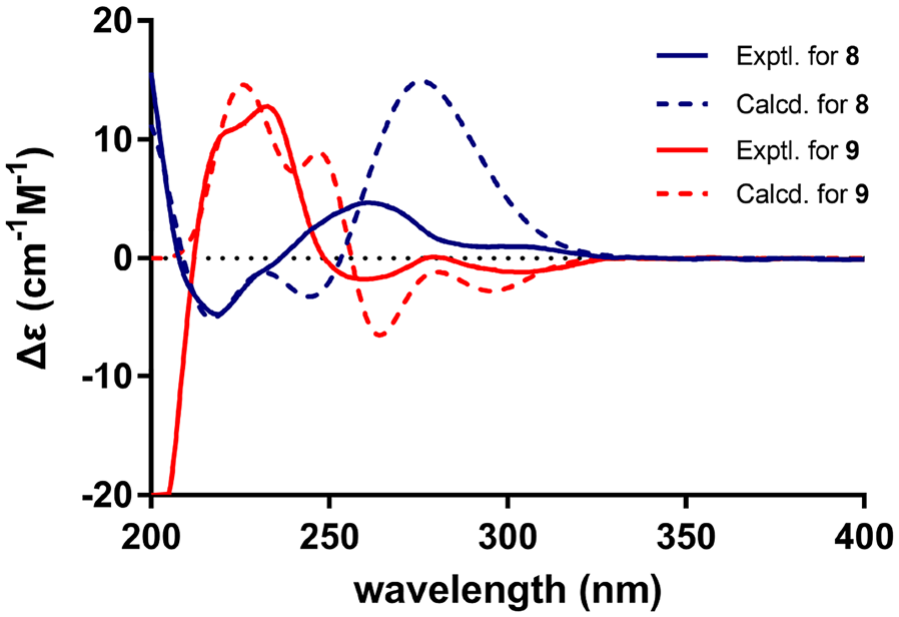
Experimental and calculated ECD spectra of **8** and **9**

Compound **8** was also isolated as yellowish oils with [α]25 D–20.0, and had a molecular formula of C_36_H_46_O_3_ according to HRESIMS at *m/z* 525.3367 [M – H]^–^ (calcd for C_36_H_45_O_3_, 525.3369). Its NMR spectroscopic data (Tables 1 and 2) was consistent with those of a known bisbakuchiol A [21], except for the chemical shifts of B ring. A set of ABX type NMR signals had disappeared in bisbakuchiol A, whereas a set of A_2_B_2_type NMR signals appeared at *δ*_H_ 7.19 (2H, br d, *J* = 8.6 Hz, H-2', 6') and 6.73 (2H, br d, *J* = 8.6 Hz, H-3', 5') in compound **8**. The configuration of **8** was elucidated through NOESY experiments (Fig. 5), where the correlation between H-8' at *δ*_H_ 3.97, and 16'-CH_3_ at *δ*_H_ 0.62 suggested their same β-orientation. The coupling constant (*J* = 7.3 Hz) between H-7' and H-8' confirmed a *trans* configuration of the two methine protons of the dioxane ring [21]. Thus, the configuration of **8**, named bisbakuchiol T, was established as 9*S*,7'*S*,8'*S*,9'*S*, which was supported by comparison of the calculated and experimental ECD curves (Fig. 6).

Compound **9** was isolated as yellowish oils with [α]25 D + 10.0, and possessed the same molecular formula, C_36_H_46_O_3_, as **8** according to the HRESIMS data (*m/z* 525.3371 [M – H]^–^). The ^1^H NMR and ^13^C NMR spectral data (Tables 1 and 2) of **9** were quite superimposable with those of compound **8**, which clearly indicated the same skeleton as that of **8**. Likely, the NOE correlation between H-7' at *δ*_H_ 4.91 and 16'-CH_3_ at *δ*_H_ 1.07, and the coupling constant (*J* = 6.1 Hz) between H-7' and H-8', indicated that the configuration of **9** was 9*S*,7'*R*,8'*R*,9'*S*, which was consistent with ECD data (Fig. 6). Therefore, the structure of compound **9**, named bisbakuchiol U, was established as shown in Fig. 1.

Compound **10** was also isolated as yellowish oils. Its HRESIMS data exhibited a sodium adduct ion at *m/z* 445.3080 [M + Na]^+^, establishing the molecular formula as C_29_H_42_O_2_. Comparison of NMR spectra of **10** (Table 3) and known bakuchiol, a side chain (3-ethenyl-3,7-dimethyl-1,6-octadienyl) and a *p*-disubstituted benzene ring of **10** were identical to that of bakuchiol. In addition, the COSY, HSQC and HMBC correlations showed the presence of 2-ethenyl-2-methyl-5-isopropanol-cyclopentan-1-ol (6-ethenyl-6-methyl-4-isopropanol-cyclopentan-5-ol) substituted group in **10**. The chemical shifts of C-3 and C-5 in **10** were shifted downfield to *δ*_C_ 124.5, suggesting that this substituted group was connected to C-4 of bakuchiol moiety by an ether linkage. The relative configuration was mainly assigned by NOESY spectrum. The signals of H-4' at *δ*_H_ 2.31 and H-5' at *δ*_H_ 4.01 showed a NOE correlation, whereas H-4' or H-5' and 11'-CH_3_ showed no correlation in its NOESY spectrum, indicating that both H-4' and H-5' were α-oriented. Therefore, the structure of compound **10**, named bakuchiol ether A, was defined as shown in Fig. 1.

Compound **11** showed a molecular formula of C_33_H_48_O_2_ on the basis of the HRESIMS ion at *m/z* 477.3713 [M + H]^+^. Similarly, compound **11** possessed a bakuchiol moiety by its' NMR data. In addition, 2D NMR correlations in **11** showed the presence of clovane-2β,9α-diol [22, 23] moiety with the exception of the resonances of C-1', C-2' to downfield shifts and C-3' and C-4' to highfield shifts. In the key HMBC spectrum (Fig. 4), the key correlation between H-2' at *δ*_H_ 4.24 and C-4 at *δ*_C_ 158.2 revealed that C-4 of bakuchiol moiety was connected to C-2' of clovane-2β,9α-diol moiety by an ether linkage. Therefore, the structure of compound **11**, named bakuchiol ether B, was defined as shown in Fig. 1.

Compound **12** was isolated as yellowish oils with [α]25 D + 30.0. It showed a molecular formula of C_33_H_48_O_2_. The COSY, HSQC and HMBC correlations showed the presences of one set of the bakuchiol signals and one set of the caryolane-1,9β-diol signals in compound **12** [23]. The chemical shifts of C-3 and C-5 were shifted downfield to *δ*_C_ 121.6 and the chemical shifts of C-1' was shifted downfield to *δ*_C_ 80.2 in **12**, suggesting that C-1' of this caryolane-1,9β-diol moiety was connected to C-4 of bakuchiol moiety by an ether linkage. Therefore, the structure of compound **12**, named bakuchiol ether C, was defined as shown in Fig. 1.

Interestingly, when the quaternary carbon from the other unit was connected to bakuchiol unit by C–O–C_4_, the chemical shifts of C-3 and C-5 would shift downfield (from115 to 121 or 123 ppm) as shown in compounds **1**, **6**, **7**, **10**, **12**, **15**, **16** and **17**. Whereas, the link by CH–O–C_4_ would not result in changes of chemical shifts at C-3 and C-5 as shown in compounds **3**, **4**, **5** and **11**. Therefore, we could infer the connection position of the dimers by the carbon chemical shifts of C-3/5 in bakuchiol unit.

NO, an unstable biological free radical, comes of *L*-arginine under the action of constitutive NO synthase (cNOS) and inducible NO synthase (iNOS). NO functions as a signaling molecule participating in neurotransmission and vasodilation. However, overproduction of NO is involved in inflammatory diseases, which can be treated by NO inhibitor. To evaluate their anti-inflammatory activities, compounds **1**–**17** (1.56–50.00 μM) were tested for inhibition effect on NO production in LPS-stimulated RAW264.7 macrophages using the Griess reaction [15]. L-NIL, a selective inhibitor of iNOS, was used for the positive control. Firstly, the cytotoxicity of these compounds at concentrations from 1.56 to 50* μM* was assessed. The MTT tests demonstrated that compounds **4** and **16** showed cytotoxicity at the concentration of 50.00 μM, whereas other compounds were not cytotoxic. The IC_50_ values of these compounds were calculated at nontoxic concentrations. As shown in Table 4, compound **1** exhibited significant inhibition of NO production with IC_50_ value at the concentration of 11.47 ± 1.57 μM, which showed no significant difference with that of L-NIL (10.29 ± 1.10 μM). Compounds **2**, **3**, **10**–**12**, **16** and **17** exhibited moderate inhibitory activities with IC_50_ values at the range of 15.98–27.80 μM. The IC_50_ values of the other compounds were more than 50.00 μM, and they showed weak inhibitory activities against NO production.

**Table 4:** Inhibition of **1**–**3**, **10**–**12** and **16**–**17** on NO production

Compound	IC_50_ (*μ*M)
**1**	11.47 ± 1.57
**2**	23.52 ± 1.82
**3**	21.43 ± 2.04
**10**	27.80 ± 1.18
**11**	26.86 ± 1.05
**12**	23.54 ± 0.82
**16**	15.98 ± 2.30
**17**	24.93 ± 1.13
L-NIL	10.29 ± 1.10

## Discussion

In our previous researches, we have obtained fourteen meroterpenoids and seventeen heterodimers of bakuchiol and evaluated their cytotoxicity [12, 13]. Further investigation on the cHE extract brought about 29 bakuchiol monomers and dimers, and their NO inhibition activities in LPS-stimulated RAW264.7 macrophages were studied. We have reported 9 monomers and 3 dimers in Chinese Traditional and Herbal Drugs [24]. In this research, seventeen bakuchiol dimmers, including twelve unpresented ones, were reported. Fortunately, a new skeleton bakuchiol dimer (**1**) was isolated, and it exhibited significant NO inhibition activities with IC_50_ value of 11.47 μM. Compounds **2**, **3**, **10**–**12**, **16** and **17** exhibited moderate inhibitory activities with IC_50_ values at the range of 15.98–27.80 μM, and other compounds showed weak inhibitory activity with IC_50_ values more than 50.00 μM.

In order to fully explore the relationship between structure and activity, results of 29 bakuchiol monomers and dimers were compared. Bakuchiol showed cytotoxicity at 12.50–50.00 μM, and exhibited weak activity with inhibitory rate of 32% at the concentration of 6.25 μM. Interestingly, structural changes at the side chain, including oxidation, cyclization and dimerization, reduced cytotoxicity. We found that the activities of uncyclized bakuchiol derivants seemed to be superior to cyclized ones. Notably, some uncyclized monomers and dimers with oxygen substitution at C-12/12′ showed stronger inhibitory activities than L-NIL, such as 12,13-dihydro-12,13-epoxybakuchiol, 12-oxobakuchiol and (12′S)-bisbakuchiol C. Among dimers, compound **1** and (12′S)-bisbakuchiol C had excellent activities, which were mostly contributed by the 6/6/5 tricyclic ketone unit and the 12,13-dihydro-12,13-dihydroxybakuchiol unit respectively. And it was worth to mention that compounds (**3**, **16** and **17**) with a psoracorylifol F unit possessed better inhibitory activities than ones (**5**–**7**) with a psoracorylifol A unit.

## Conclusion

Seventeen bakuchiol dimers (**1**–**17**), including 12 undescribed dimers and 5 known compounds, were isolated from the fruits of *Psoralea corylifolia* L. and their structure were identified by spectral methods and X-ray single-crystal diffraction. Bisbakuchiol M (**1**), whose other bakuchiol unit was cyclized to form a 6/6/5 tricyclic system, was a new skeleton compound. And the plausible biosynthetic pathway of bisbakuchiol M was proposed. Their inhibition on NO production in LPS-stimulated RAW264.7 macrophages were evaluated by the Griess reaction. Compounds **2**, **3**, **10**–**12**, **16** and **17** exhibited inhibitory activities, and the inhibition of compound **1** was equal to that of L-NIL. Their structure–activity relationship was discussed, showed that uncyclized monomers and dimers with oxygen substitution at C-12/12′ showed strong inhibitory activities. And carbonyl units contributed to enhanced activities. These findings suggested that *Psoraleae Fructus* provided natural anti-inflammatory constituents and were of great significance in the design for anti-inflammatory drug.

## Supplementary Information

**Additional file 1.** **Fig. S1.** MRM chromatogram (A: reference solution, B: test solution) for compounds 1–3, 6–9, and 13–17.

## Data Availability

All data included in this article are available from the corresponding author upon request.

## Abbreviations

TCM: Traditional Chinese medicine
NO: Nitric oxide
IC_50_: Half maximal inhibitory concentration
CC: Open column chromatography
TLC: Thin layer chromatography
RP-SP-HPLC: Reversed phase semi-preparative HPLC
PE: Petroleum ether
cHE: Cyclohexane
EtOAc: Ethyl acetate
DMEM: Dulbecco's modified Eagle's medium
FBS: Fetal bovine serum
CHCl_3_: Chloroform
BuOH: Normal-butanol
MTT: 3-(4,5-Dimethyl-2-thiazolyl)-2,5-diphenyl-2H-tetrazolium bromide
LPS: Lipopolysaccharide
DMSO: Dimethylsulfoxide
L-NIL: L-N6-(1-iminoethyl)-lysine
