# Supplementary figures and images for: New bakuchiol dimers from Psoraleae Fructus and their inhibitory activities on nitric oxide production
Source: Chin Med. 2021 Oct 7;16:98. doi: 10.1186/s13020-021-00499-y (PMC8499495; doi:10.1186/s13020-021-00499-y)

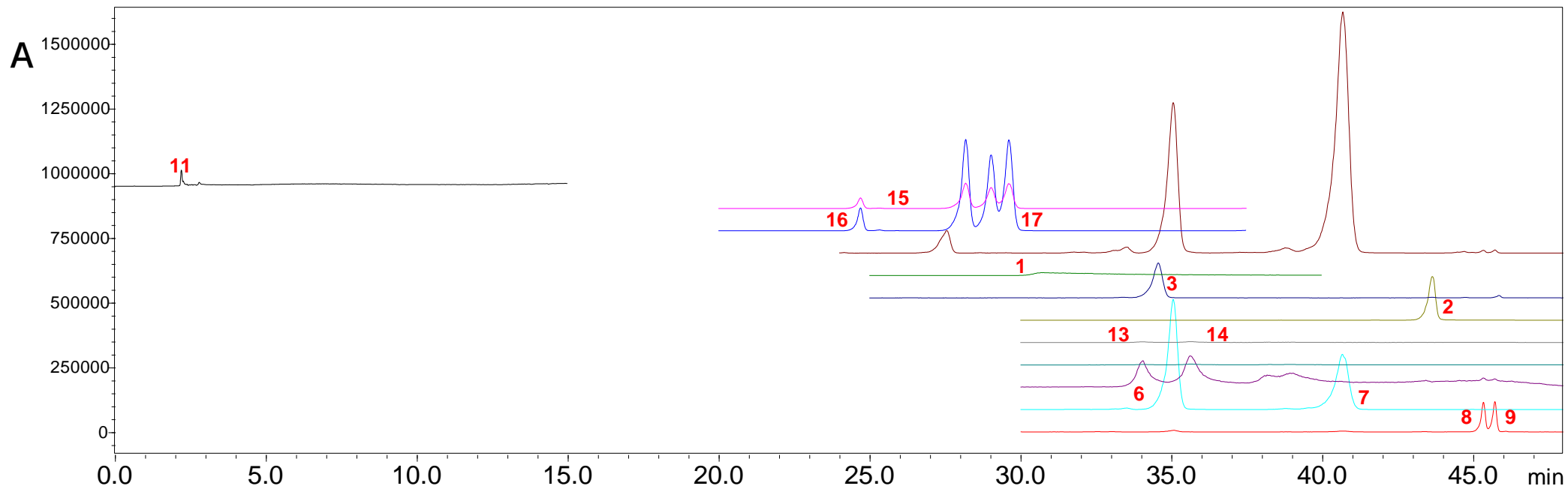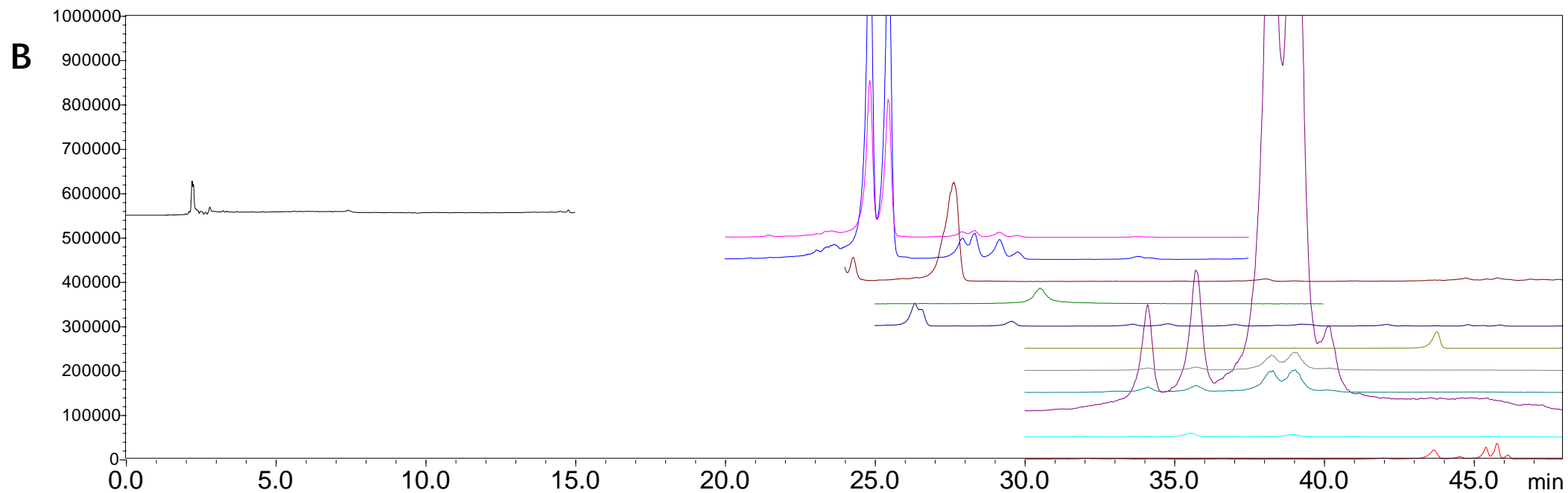

Supplement: Supplementary file 1 — Additional file 1. Fig. S1. MRM chromatogram (A: reference solution, B: test solution) for compounds 1–3, 6–9, and 13–17. [file 13020_2021_499_MOESM1_ESM.pdf]
